# The Rheumatic Diseases (So-Called), with Original Suggestions for More Clearly Defining Them

**Published:** 1890-06

**Authors:** 


*The Rheumatic Diseases (so-called), with original suggestions for more clearly defining them.* By HUGH LANE, L.R.C.P. Ed., M.R.C.S. Eng., Honorary Physician to the Eastern Dispensary, Bath; and CHARLES J. GRIFFITHS, L.R.C.P. Lond., M.R.C.S. Eng., Resident Medical Officer to the Royal Mineral Water Hospital, Bath. London: J. & A. CHURCHILL. 1890.

We are glad to welcome anything tending to clear away the fog which enshrouds the chaotic region intervening between acute rheumatism on the one hand and acute gout on the other. The immense field of observation afforded by the large collection of chronic rheumatic cases in and around the Bath Mineral Water Hospital has enabled the authors to analyse critically some 1200 cases, and the results of their analysis are given in this book, which is a purely clinical one and must not be criticised from the point of view of pathology. Four classes of chronic joint-disease are described:—

1. Chronic rheumatism.
2. Chronic rheumatic arthritis.
3. Rheumatoid arthritis.
4. Chronic gout.

And the most characteristic feature of the book is the attempt to differentiate between what is true rheumatoid arthritis and what is not, the main points being epitomised in the following table:—

| *Rheumatoid Arthritis.* | *Rheumatic Arthritis.* |
|---|---|
| 1. Nervous disease due to debilitating causes. | 1. Following rheumatism always. |
| 2. Its last stage is osteo-arthritis. | 2. Has no connection with it. |
| 3. Symptoms constitutional as well as local. | 3. More confined to joints. |
| 4. Joints most used are first affected, smaller joints first, running centripetally. Temporo-maxillary joints often affected. Symmetry of joints affected noticeable. | 4. Large joints often first affected, running centrifugally, and chiefly joints attacked in previous acute rheumatism. Temp.-max. joints never affected. Not so often symmetrical. |
| 5. Swelling typical, more or less fusiform, and with appearance of effusion. | 5. Swelling as if solid enlargement of normal joint. |
| 6. Deformity varying. | 6. Greater tendency to fixation of joints in flexed position. Deformity in fingers is fixation in position of extreme flexion. |
| 7. Anæmia, early and constant. | 7. Later, and less intense. |
| 8. Neurotic symptoms, sweating, headache, tingling, numbness, pigmentation of skin, &c. | 8. Absent. |
| 9. Rarely subacute attacks. | 9. Greater tendency. |
| 10. Heart normal, but rapid in action. | 10. Often diseased. |
| 11. Hard rapid pulse. | 11. Pulse varies with state of heart. |
| 12. Reflexes normal or subnormal. | 12. Increased. |
| 13. Muscular atrophy concurrent with, and often previous to, joint affection, and small muscles chiefly. | 13. Muscular atrophy subsequent to joints being affected, often large muscles first. |
| 14. Any age. | 14. Adults, and mostly over middle age. |

We believe that still more may yet be done in the diagnosis and treatment of these rheumatoid conditions, and that the authors of this book will be called upon for further work in elaborating and improving the volume as subsequent editions are called for.

